# Supplementary material for: Food Tracking Perspective: DNA Metabarcoding to Identify Plant Composition in Complex and Processed Food Products
Source: Genes (Basel). 2019 Mar 25;10(3):248. doi: 10.3390/genes10030248 (PMC6470991; doi:10.3390/genes10030248)
Supplement: Supplementary file 1 [file genes-10-00248-s001.zip › genes-452785-supplementary_revised/TableS1_S2_S3_S4.docx]

| fruit mixtures | ananas (*Ananas comosus)* | avocado (*Persea americana*) | dragon fruit (*Hylocereus undatus*) | mango (*Mangifera indica*) | papaya  (*Carica papaya*) |
| --- | --- | --- | --- | --- | --- |
| mix 1 | 1 | 1/10 | 1/25 | 1/50 | 1/100 |
| mix 2 | 1/100 | 1 | 1/10 | 1/25 | 1/50 |
| mix 3 | 1/50 | 1/100 | 1 | 1/10 | 1/25 |
| mix 4 | 1/25 | 1/50 | 1/100 | 1 | 1/10 |
| mix 5 | 1/10 | 1/25 | 1/50 | 1/100 | 1 |

**Table S1**. **DNA proportions of fruits used for fruit mixtures.** Five different artificial fruit mixtures were prepared combining different fruits. Each column reports the proportions of DNA used for each fruit to constitute the mock fruit mixtures.

| fruit mixtures | ananas (*Ananas comosus)* | avocado (*Persea americana*) | dragon fruit (*Hylocereus undatus*) | mango (*Mangifera indica*) | papaya  (*Carica papaya*) |
| --- | --- | --- | --- | --- | --- |
| mix 1 | 7086773.556 | 7202515.723 | 418367.5116 | 3022749.092 | 8031429.168 |
| mix 2 | 708677.3556 | 720251.5723 | 41836.75116 | 302274.9092 | 803142.9168 |
| mix 3 | 283470.9422 | 288100.6289 | 16734.70046 | 120909.9637 | 321257.1667 |
| mix 4 | 141735.4711 | 144050.3145 | 8367.350232 | 60454.98183 | 160628.5834 |
| mix 5 | 70867.73556 | 72025.15723 | 4183.675116 | 30227.49092 | 80314.29168 |

**Table S2**. **DNA copies of *trn*L gene measured with qPCR.** Five different artificial fruit mixtures were prepared combining different fruits. Each column reports the proportions of DNA (expressed as the DNA copies of *trn*L gene) used for each fruit to constitute the mock fruit mixtures. Concentrations are measured using qPCR.

| **sample_name** | **seq_barcode_F** | **seq_barcode_R** |
| --- | --- | --- |
| Saffron | tagctagt | atgatcgc |
| Flavoured_tea | agcacagt | atgatcgc |
| Vegetable_stock_cube | gatgatct | atgatcgc |
| Curry | tagctagt | ctgcgtac |
| Pureed_deep-frozen_vegetables | gatgatct | ctgcgtac |
| Food_supplement | acatgtgt | gactgatg |
| Mock_herbal_mix | ctgcgtac | ctgcgtac |
| mix_1 | acacacac | acacacac |
| mix_2 | gtgtacat | acacacac |
| mix_3 | tatgtcag | acacacac |
| mix_4 | tagtcgca | acacacac |
| mix_5 | tactatac | acacacac |
| mix_1 | acacacac | actagatc |
| mix_2 | gtgtacat | actagatc |
| mix_3 | tatgtcag | actagatc |
| mix_4 | tagtcgca | actagatc |
| mix_5 | tactatac | actagatc |
| mix_1 | cgctctcg | acacacac |
| mix_2 | gtcacgtc | acacacac |
| mix_3 | gactgatg | acacacac |
| mix_4 | agactatg | acacacac |
| mix_5 | gcgtcagc | acacacac |
| mix_1 | cgctctcg | actagatc |
| mix_2 | gtcacgtc | actagatc |
| mix_3 | gactgatg | actagatc |
| mix_4 | agactatg | actagatc |
| mix_5 | gcgtcagc | actagatc |
| mix_1 | gtacgact | agactatg |
| mix_2 | acgacgag | agactatg |
| mix_3 | catcagtc | agactatg |
| mix_4 | atcagtca | agactatg |
| mix_5 | tctactga | agactatg |
| mix_1 | gtacgact | acgacgag |
| mix_2 | acgacgag | acgacgag |
| mix_3 | catcagtc | acgacgag |
| mix_4 | atcagtca | acgacgag |
| mix_5 | tctactga | acgacgag |
| mix_1 | agcgacta | agactatg |
| mix_2 | actctgct | agactatg |
| mix_3 | atatagcg | agactatg |
| mix_4 | ctatgcta | agactatg |
| mix_5 | tcgcgctg | agactatg |
| mix_1 | agcgacta | acgacgag |
| mix_2 | actctgct | acgacgag |
| mix_3 | atatagcg | acgacgag |
| mix_4 | ctatgcta | acgacgag |
| mix_5 | tcgcgctg | acgacgag |

**Table S3**. **List of barcodes used for Illumina sequencing**
